# Detection of toxigenic *Clostridium difficile* colonization in patients admitted to the hospital for chemotherapy or haematopoietic cell transplantation

**DOI:** 10.1099/jmm.0.000774

**Published:** 2018-06-04

**Authors:** John L. Vaughn, Joan-Miquel Balada-Llasat, Misty Lamprecht, Ying Huang, Mirela Anghelina, Zeinab El Boghdadly, Karen Bishop-Hill, Rachel Childs, Preeti Pancholi, Leslie A. Andritsos

**Affiliations:** ^1^​ Division of Hematology, The Ohio State University Wexner Medical Center, Columbus, OH, USA; ^2^​ Department of Pathology, The Ohio State University Wexner Medical Center, Columbus, OH, USA; ^3^​ Comprehensive Cancer Center, The Ohio State University Wexner Medical Center, Columbus, OH, USA; ^4^​ Division of Infectious Diseases, The Ohio State University Wexner Medical Center, Columbus, OH, USA; ^5^​ Nursing Research, The Ohio State University Wexner Medical Center, Columbus, OH, USA

**Keywords:** *Clostridium difficile*, chemotherapy, haematopoietic cell transplantation, colonization

## Abstract

Increasing evidence suggests that asymptomatic carriers are an important source of healthcare-associated *Clostridium difficile* infection. However, it is not known which test for the detection of *C. difficile* colonization is most sensitive in patients with haematological malignancies. We performed a prospective cohort study of 101 patients with haematological malignancies who had been admitted to the hospital for scheduled chemotherapy or haematopoietic cell transplantation. Each patient provided a formed stool sample. We compared the performance of five different commercially available assays, using toxigenic culture as the reference method. The prevalence of toxigenic *C. difficile* colonization as determined by toxigenic culture was 14/101 (14 %). The Cepheid Xpert PCR *C. difficile*/Epi was the most sensitive test for the detection of toxigenic *C. difficile* colonization, with 93 % sensitivity and 99 % negative predictive value. Our findings suggest that the Xpert PCR *C. difficile*/Epi could be used to rule out toxigenic *C. difficile* colonization in this population.

## Full-Text

*Clostridium difficile* is an important cause of infectious diarrhoea in patients with haematological malignancies who are admitted to the hospital for chemotherapy or haematopoietic cell transplantation (HCT) [1, 2]. These patients are at increased risk of developing *C. difficile* infection (CDI) compared to non-oncology patients, and they are more likely to suffer adverse outcomes from the infection [2, 3]. A study from one transplant centre identified HCT patients as having a ninefold higher rate of CDI than non-oncology patients at the same institution, and a 1.4× greater rate than other oncology patients [2]. Due to the profound and often prolonged duration of immunosuppression experienced by patients with haematological malignancies, the risk for the development of complications associated with CDI is high [3, 4]. For example, the development of CDI in patients who have undergone allogeneic HCT has been associated with increased risk of graft-versus-host disease and non-relapse mortality [1, 3].

Asymptomatic colonization of patients with *C. difficile* is known to precede infection [5–8]. However, the most sensitive method for detecting toxigenic *C. difficile* colonization in formed stool is unknown. A variety of tests are available for the detection of toxigenic *C. difficile* in diarrhoeal samples [9, 10]. Tests targeting *C. difficile* toxins are commonly used. However, changes in the temperature and chemical composition of the faeces may affect toxin stability, potentially yielding false-negative test results [10]. Tests detecting the enzyme glutamate dehydrogenase (GDH) are also available but have low specificity, since GDH is highly conserved in all isolates of *C. difficile*, including non-toxigenic strains [10]. Nucleic acid amplification tests (NAATs) that detect the presence of *C. difficile* gene targets such as tcdA, tcdB and 16S ribosomal RNA are available and have high sensitivity [10].

There are two widely used reference methods for the detection of toxigenic *C. difficile*: toxigenic culture (TC) and cell cytotoxicity assay (CCTA) [9–11]. Although both methods are widely used, they detect different targets: the CCTA detects the presence of *C. difficile* toxins, and TC detects the presence of *C. difficile* with the potential to produce toxin [11]. Hence, the poor performance of a test for the detection of toxigenic *C. difficile* may be due to the reference method used and not the performance of the test itself [11]. The turnaround time for TC is approximately 3 days, which limits its use in routine clinical practice [9]. No tests are approved by the United States Food and Drug Administration (FDA) for testing on formed stool.

We performed a prospective cohort study of patients with haematological malignancies admitted for scheduled chemotherapy or HCT at our institution. Patients were included if they were aged 18 years or older and scheduled for elective admission for chemotherapy or HCT (including both autologous and allogeneic HCT). Patients were excluded if they had experienced diarrhoea due to CDI in the past 30 days; if they had experienced diarrhoea of unknown aetiology; if they were admitted on an unscheduled basis for the treatment of acute illness; or if their anticipated length of stay was less than 72 h.

At the time of hospital admission for scheduled chemotherapy or HCT, a formed stool specimen was collected from each patient in a sterile collection vial. All samples were freshly collected and sent to the microbiology laboratory for testing. The stool samples were tested with six different methods: Simplexa *C. difficile* PCR Universal Direct kit (Diasorin, Saluggia, Italy), Xpert PCR *C. difficile*/Epi (Cepheid, Sunnyvale, CA, USA), C. diff Quik Chek Complete GDH (Alere, Waltham, MA, USA), C. diff Quik Chek Complete Toxin A/B (Alere, Waltham, MA, USA), LEUKO EZ VUE ELISA test (Alere, Waltham, MA, USA) and TC.

The Simplexa *C. difficile* PCR Universal Direct kit is a real-time polymerase chain reaction (PCR) assay for the *in vitro* qualitative direct detection of the toxin B gene (tcdB) of *C. difficile*. The Xpert *C. difficile*/Epi PCR assay is a multiplex real-time PCR that detects tcdB, the binary toxin gene (cdt) and the tcdC gene deletion at nt 117. The C. diff Quik Chek Complete includes tests for both GDH antigen and *C. difficile* toxins A and B in faecal specimens. The LEUKO EZ VUE ELISA test is a rapid lateral flow cassette test that is used to detect elevated levels of lactoferrin, a marker of faecal leukocytes. Although the LEUKO EZ VUE ELISA test does not detect toxigenic *C. difficile*, we included the test to determine whether elevated levels of lactoferrin also occur in patients with toxigenic *C. difficile* colonization.

TC was performed by plating stool specimens onto prereduced cycloserine/cefoxitin/fructose agar media (CCFA-VA formulation, Remel, Lenexa, KS, USA) with alcohol shock pretreatment [12]. Plates were incubated anaerobically using the anaerobe chamber (Bactron IV, Sheldon Manufacturing, Cornelius, OR, USA) at 35 °C for up to 5 days before a final interpretation of a negative result. *C. difficile* was identified by matrix-assisted laser desorption/ionization-time of flight (MALDI-TOF) mass spectrometry.

After the *C. difficile* colonies were identified, the isolates were grown for 24 h in anaerobic Brucella broth (Remel), and the supernatant was passed through a 0.22 µm filter (Spin-X centrifuge tube filter; Millipore, Billerica, MA, USA). We added 50 µl of filtrate to skin fibroblast cells (96-well microtitre plate, Diagnostics Hybrids, Athens, OH, USA), which were then incubated for 48 h at 37 °C, 5 % CO_2_. In order to control for nonspecific toxicity, a second well was inoculated with both the supernatant and 50 µl of *C. difficile* goat antitoxin (TECHLAB, Blacksburg, VA, USA). The cells were incubated at 37 °C and checked for cytopathic effect (CPE) at 6, 22, 30 and 48 h. A positive result was defined as the presence of CPE in at least 50 % of the cell monolayer and no CPE in the tube inoculated with the antitoxin.

Patients’ demographic and baseline clinical characteristics were described using summary statistics. Fisher’s exact test was used to compare categorical variables between those who tested positive and those who tested negative by TC, and the Wilcoxon rank sum test was used for continuous variables. Since only 14 cases of positive *C. difficile* colonization were detected through TC, univariable logistic regression analysis was used to identify risk factors for *C. difficile* colonization in an exploratory manner. Sensitivity and specificity with 95 % binomial exact confidence interval (CI) for five tests were estimated.

A total of 114 patients were recruited for participation between 1 April 2016 and 30 November 2016. Thirteen patients either withdrew consent or did not provide a stool sample, so the final analysis included 101 stool samples. The median age of the study cohort was 60 (range, 19–84) years. As shown in Table 1, the most common disease type was lymphoma (*n*=48, 48 %) followed by multiple myeloma (*n*=24, 24 %), leukaemia (22 %), MDS (*n*=5, 5 %), AL amyloidosis (*n*=1, 1 %) and aplastic anemia (*n*=1, 1 %). The most common reason for admission was scheduled chemotherapy (*n*=49, 49 %), followed by autologous HCT (*n*=34, 34 %) and allogeneic HCT (*n*=18,18 %). The median (range) length of hospital stay was 13 (2–57) days.

**Table 1.** Characteristics of patients with haematological malignancies admitted to the hospital for chemotherapy or HCT

Characteristic	Overall (*n*=101)	Toxigenic culture positive (*n*=87)	Toxigenic culture negative (*n*=14)	*P-*value
Age [median (range)], years	60 (19–84)	59 (19–84)	60.5 (29–81)	0.42
Sex				0.57
Female	48 (48)	40 (46)	8 (57)	
Male	53 (52)	47 (54)	6 (43)	
Race				1.00
White	88 (91)	76 (90)	12 (92)	
Black	9 (9)	8 (10)	1 (8)	
Unknown	4	3	1	
Disease				0.43
AL amyloidosis	1 (1)	1 (1)	0 (0)	
Aplastic anaemia	1 (1)	1 (1)	0 (0)	
Leukaemia	22 (22)	17 (20)	5 (36)	
Lymphoma	48 (48)	40 (46)	8 (57)	
MDS	5 (5)	5 (6)	0 (0)	
Multiple myeloma	24 (24)	23 (26)	1 (7)	
Reason for admission				0.17
Allogeneic HCT	18 (18)	16 (18)	2 (14)	
Auto HCT	34 (34)	32 (37)	2 (14)	
Chemotherapy	49 (49)	39 (45)	10 (71)	
ANC [median (range)], 1000 mm^−3^	59.8 (0–92.9)	61.9 (0–92.9)	52.2 (2.2–89.7)	1.00
Albumin [median (range)], mg dl^−1^	4 (2.7–4.8)	4 (2.7–4.8)	4 (3.5–4.5)	0.84
Serum creatinine [median (range)], mg dl^−1^	0.8 (0.4–6.1)	0.8 (0.4–6.1)	0.8 (0.6–1.6)	0.85
WBC [median (range)], 1000 mm^−3^	6 (0.5–259)	5.4 (0.5–259)	6.9 (4.2–22.4)	0.02
Antibiotics in past 30 days				1.00
No	58 (57)	50 (57)	8 (57)	
Yes	43 (43)	37 (43)	6 (43)	
Prior CDI				0.14
No	100 (99)	87 (100)	13 (93)	
Yes	1 (1)	0 (0)	1 (7)	
Immunosuppression in past 30 days*				0.008
No	97 (96)	86 (99)	11 (79)	
Yes	4 (4)	1 (1)	3 (21)	
Treatment with proton pump inhibitor				0.39
No	60 (59)	50 (57)	10 (71)	
Yes	41 (41)	37 (43)	4 (29)	
Treatment with histamine antagonist				0.61
No	92 (91)	80 (92)	12 (86)	
Yes	9 (9)	7 (8)	2 (14)	

Data are presented as *n* (%) unless otherwise specified.

Abbrevations: CD, *Clostridium* *difficile*; CDI, *Clostridium* *difficile* infection; HCT, haematopoietic cell transplantation; WBC, white blood cell.

*Immunosuppression includes tacrolimus, mycophenolate mofetil and rituximab.

The prevalence of toxigenic *C. difficile* colonization as determined by TC was 14/101 {14 %, [95 % confidence interval (CI): 8–22 %]}. Compared with non-colonized patients, colonized patients were more likely to have received immunosuppressive therapy prior to admission and had slightly higher median white blood cell count (Table 1). However, in a univariable regression model, none of these factors were independent predictors of *C. difficile* colonization. The results of the univariable logistic regression analysis are shown in Table 2.

**Table 2.** Univarable logistic regression analysis for patients colonized with toxigenic *C. difficile* according to toxigenic culture

Characteristic	Odds ratio (95 % CI)	Likelihood ratio *P*-value
Age, 1 year increase	1.02 (0.97–1.07)	0.46
Sex, male vs female	0.64 (0.20–2.00)	0.44
Race, white vs black	1.26 (0.15–11.02)	0.83
Allogenic HCT	0.49 (0.10–2.48)	0.39
Auto HCT	0.24 (0.05–1.19)	0.08
ANC, 1000 mm^−3^	1.00 (0.98–1.02)	0.86
Albumin, mg dl^−1^	1.09 (0.28–4.31)	0.90
Serum creatinine, mg dl^−1^	0.80 (0.26–2.41)	0.66
WBC, 1000 mm^−3^ (twofold increase)	1.51 (0.94–2.41)	0.08
Antibiotics in past 30 Days, yes vs no	1.01 (0.32–3.17)	0.98
Immunosuppression, yes vs no*	2.15 (0.21–22.3)	0.55
Treatment with proton pump inhibitor, yes vs no	0.54 (0.16–1.86)	0.31
Treatment with histamine antagonist, yes vs no	1.91 (0.35–10.27)	0.47

Abbreviations: ANC, absolute neutrophil count; CD, *Clostridium* *difficile*; CI, confidence interval; WBC, white blood cell.

*Immunosuppression includes tacrolimus, mycophenolate mofetil and rituximab.

Table 3 shows the positive and negative test results for each testing method compared to TC. The Xpert PCR *C. difficile*/Epi had the highest sensitivity of 93 % (95 % CI: 66–100 %) for the detection of *C. difficile* colonization. Only one patient was positive for *C. difficile* colonization by TC but negative by the Xpert PCR *C. difficile*/Epi. The specificity of the Xpert PCR *C. difficile*/Epi was relatively low at 92 % (95 % CI: 84–97 %). A total of seven patients were positive by the Xpert PCR *C. difficile*/Epi but negative by TC, which reduced the specificity of the Xpert PCR test in our analysis. The Simplexa *C. difficile* PCR Universal Direct kit also had seven false positives, but the patients were not the same (only four patients overlapped).

**Table 3.** Positive and negative test results for toxigenic *C. difficile* compared to the gold standard of toxigenic culture

Test	TC Neg	TC pos	Sensitivity [% (95 % CI)]	Specificity [% (95 % CI)]	PPV [% (95 % CI)]	NPV [% (95 % CI)]
Xpert PCR			93 (66–100)	92 (84–97)	65 (41–85)	99 (93–100)
Negative	80	1				
Positive	7	13				
Simplexa PCR			79 (49–95)	92 (84–97)	61 (36–83)	96 (90–99)
Negative	80	3				
Positive	7	11				
GDH			71 (42–92)	83 (73–90)	40 (21–61)	95 (87–99)
Negative	72	4				
Positive	15	10				
Toxin A/B			29 (8–58)	100 (96–100)	100 (40–100)	90 (82–95)
Negative	87	10				
Positive	0	4				
Lactoferrin			36 (13–65)	77 (67–85)	20 (7–41)	88 (79–94)
Negative	67	9				
Positive	20	5				

Abbreviations: GDH, glutamate dehydrogenase; NPV, negative predictive value; PCR, polymerase chain reaction; PPV, positive predictive value; TC Neg, toxigenic culture-negative; TC Pos, toxigenic culture-positive.

From our study data, the test with the lowest sensitivity was the *C. diff* Quik Chek Complete Toxin A/B, which was only 29 % (95 % CI: 8–58 %) sensitive. However, the toxin test had the highest specificity of 100 % (95 % CI: 96–100), since all four positive results were also positive by TC. The low sensitivity of the toxin test may have been due to the reference method used, since TC does not measure toxin directly [11]. Among the 14 patients who tested positive by TC, only 1 patient (7 %) developed CDI during his hospitalization, and his formed stool tested positive by TC and Xpert PCR *C. difficile*/Epi prior to the development of CDI. None of the other methods were positive for the patient who developed CDI.

In our study of patients with haematological malignancies admitted to the hospital for chemotherapy or HCT, we found that 14 % of patients were colonized with toxigenic *C. difficile* on hospital admission. Our findings are consistent with those of other recently published studies [6, 13, 14]. Bruminhent *et al*. [14] found that 10.7 % of HCT patients admitted to the hospital were colonized with toxigenic *C. difficile*. Similarly, Cannon *et al*. [13] found that 9.3 % of haematology/oncology patients admitted to the hospital were colonized with toxigenic *C. difficile*. However, to the best of our knowledge, this is the first study to compare different commercially available testing methods that could be used to screen for toxigenic *C. difficile* colonization in this population.

It is not known which method is optimal for testing patients for *C. difficile* colonization. In patients who are suspected of having CDI based on clinical symptoms (e.g. diarrhoea), the most recent guidelines from The Infectious Diseases Society of America recommend using a NAAT alone or a multistep testing algorithm [10]. However, there are no comparable guidelines for testing asymptomatic patients with formed stool. In our study, the Xpert PCR *C. difficile*/Epi had the highest sensitivity and negative predictive value for *C. difficile* colonization, with a quick turnaround time of 45 min. Thus, our results suggest that NAATs may be the preferred method for testing for *C. difficile* colonization in asymptomatic patients on hospital admission. It is possible that some of the false-positive results for the NAATs in our study were actually false-negative culture results. Regardless, the low positive predictive values of NAATs in this setting may necessitate the use of a multistep testing algorithm for confirmation of colonization [15].

Increasing evidence suggests that asymptomatic carriers are a source of healthcare-associated CDI [16, 17]. For example, in a study of 56 patients with healthcare-associated CDI, Curry *et al*. [17] found that 16 (29 %) cases were tied to asymptomatic carriers. Based in part on those findings, Longtin *et al.* [18] performed a controlled study to determine the effect of detecting and isolating *C. difficile* asymptomatic carriers at hospital admission on the incidence of healthcare-associated CDI. The authors found that detecting and isolating *C. difficile* carriers was associated with a significant decrease in the incidence of healthcare-associated CDI. Given the limited evidence, it is unclear if isolating colonized patients has an impact on the incidence of healthcare-associated CDI. Additional larger studies are needed to further investigate the clinical implications and cost-effectiveness of such an approach.

Our study has several limitations. It was single-institutional, so our findings may not be generalizable to other settings. Additionally, our cohort was limited to patients with diagnoses of haematological malignancies, so our results may not reflect the majority of general medicine patients or patients admitted to the hospital for non-chemotherapy or HCT indications. Our use of TC as the reference method instead of CCTA may have caused us to underestimate the performance of some of the tests. However, despite its limitations, our study provides novel data comparing different commercially available testing methods to screen asymptomatic patients for toxigenic *C. difficile* colonization. The identification of colonization will allow providers to explore potential preventive strategies for prophylaxis and preemptive treatment for CDI in this population, as well as for the implementation of isolation strategies to prevent nosocomial transmission of infection.

In conclusion, we found that 14 % of the patients in our cohort were colonized with *C. difficile* on hospital admission. The Cepheid Xpert PCR *C. difficile*/Epi was the most sensitive test for the detection of *C. difficile* colonization, with a sensitivity of 93 %. Its high sensitivity and quick turnaround time suggest that it could be used to screen patients for *C. difficile* colonization on hospital admission. Future randomized controlled studies will help to determine whether isolating patients colonized with *C. difficile* is an effective way to reduce healthcare-associated CDI.
